# Draft Genome Sequence of *Bacteroidales* Strain TBC1, a Novel Isolate from a Methanogenic Wastewater Treatment System

**DOI:** 10.1128/genomeA.01168-15

**Published:** 2015-10-08

**Authors:** Dieter M. Tourlousse, Norihisa Matsuura, Liwei Sun, Mayu Toyonaga, Kyohei Kuroda, Akiko Ohashi, Rodrigo Cruz, Takashi Yamaguchi, Yuji Sekiguchi

**Affiliations:** a Biomedical Research Institute, National Institute of Advanced Industrial Science and Technology (AIST), Tsukuba, Ibaraki, Japan; b School of Energy & Environment, Southeast University, Nanjing, Jiangsu, China; c Department of Civil and Environmental Engineering, Nagaoka University of Technology, Nagaoka, Niigata, Japan; d EPAS International NV, Ghent, Belgium

## Abstract

We report here the draft genome sequence of *Bacteroidales* strain TBC1, isolated from a methanogenic wastewater treatment system. The draft genome has a size of 4,514,407 bp and a G+C content of 46.7%. The predicted genomic content provides the basis for characterizing the metabolism and ecological strategies of strain TBC1.

## GENOME ANNOUNCEMENT

In addition to their prevalence in host-associated habitats, such as the gut (1, 2), members of the order *Bacteroidales* are also abundant in a range of other organic-rich anoxic environments, including anaerobic waste/wastewater treatment systems (3, 4). A vast diversity of environmental *Bacteroidales* lineages, including novel taxa at the family level, remain to be cultured and are hence poorly understood. Given that *Bacteroidales* may represent core members of various anoxic/anaerobic ecosystems (2, 4), continued efforts toward understanding their metabolic versatility and ecology are therefore warranted.

We previously isolated a novel bacterium, designated strain TBC1, from a methanogenic wastewater treatment system operated in Hungary. Phylogenetic analysis of its 16S rRNA gene (DDBJ/EMBL/GenBank accession no. LC049960) indicated that strain TBC1 belongs to a hitherto uncultured cluster within the *Bacteroidales*, named BA008 in the Greengenes database (May 2013 release [5]). Strain TBC1 shares <86% sequence similarity (as determined by BLAST) with species in the Living Tree Project database (release 121 [6]); its closest relative (85.9% similarity) was the soil bacterium *Mucilaginibacter boryungensis* (accession no. HM061614). Here, we generated a high-quality draft genome sequence of strain TBC1 to support the further characterization of its metabolism and ecophysiology.

Generation of the TBC1 draft genome was performed as follows. Paired-end (300- to 1,000-bp inserts, Nextera XT indexed) and Nextera mate-pair (1- to 14-kbp insert) libraries were generated and sequenced on an Illumina MiSeq instrument using V2 chemistry (2 × 250-bp reads). The expected coverages were 40× and 10× for the paired-end and mate-pair libraries, respectively. In terms of read processing, raw reads were overlapped with SeqPrep using default settings, including the removal of sequencing adapters. Reads that failed to merge were quality trimmed and filtered using Nesoni version 0.112. Both merged and processed single reads were assembled using SPAdes version 2.5.0 (7), followed by manual curation of the assembly (8). The genome was annotated using the Integrated Microbial Genomes pipeline (9).

The final high-quality draft assembly has a coverage of 50× and consists of 15 contigs resolved into 2 scaffolds. The size of the draft genome is 4,514,407 bp, and the mean G+C content is 46.7%. Phyla-AMPHORA (10) identified 211 (out of 215) *Bacteroidetes*-specific phylogenetic marker genes, which verified that the genome is nearly complete. Annotation predicted 3,402 protein-coding genes and 53 RNA genes in the genome; approximately 75% of the proteins have a predicted function. Interestingly, the genome harbors three copies of the superoxide dismutase gene, suggesting that strain TBC1 is tolerant to oxidative stress. The presence of a large number genes encoding sensory proteins furthermore underscored that the strain may be highly responsive to environmental cues. We anticipate that the genome of strain TBC1 will advance our knowledge on the metabolic and ecological strategies employed by previously uncultured lineages of the order *Bacteroidales*.

### Nucleotide sequence accession numbers.

This whole-genome shotgun project has been deposited at DDBJ/EMBL/GenBank under the accession no. BBYI00000000. The version described in this paper is BBYI01000000.
